# Self-awareness in fish?

**DOI:** 10.1073/pnas.2303377120

**Published:** 2023-05-01

**Authors:** Alain Morin

**Affiliations:** ^a^Department of Psychology, Mount Royal University, Calgary, AB T3E 6K6, Canada

Kohda et al. (1) present results suggesting that cleaner fish can recognize a photograph of themselves based on introspection of a mental image of their face (“autoscopic” image), which they take as evidence of private self-awareness. In my opinion, the report suffers from several limitations that cast doubt on the conclusions reached.

A first issue pertains to the following reasoning: “… recognition of the self in a photograph cannot be achieved via a kinesthetic visual-matching mechanism, as photographs are motionless, and self-recognition must therefore occur by a mental image of the self ” (p. 1). This is questionable. There still could be other unknown underlying mechanisms no one thought about yet, which could account for the results, and which would not entail imagery. As a matter of fact, there is no objective evidence presented in the paper that the fish created and used an autoscopic image to pass the self-recognition test. This is an assumption made by the authors to make sense of their results.

A second problem is that even if we were to accept that the experimental fish formed and used an autoscopic image, it would only show that fish may possess one single, tiny, aspect of private self-awareness. It would remain uninformative regarding other important aspects of self-reflection—namely, access to internal states and experiences such as emotions, goals, sensations, memories, thoughts, etc.

A third observation refers to the authors’ concluding remarks: “… It has, however, also been assumed that language, particularly inner speech, is essential for higher levels of self-processing, including the development of mental states or private self-awareness… Our results therefore demonstrate the need to reconsider the assumed importance of inner speech for higher cognitive functions, including self-awareness” (p. 6). What “higher cognitive functions” were investigated in the study? The authors claim autoscopic images, but in practicality, they measured self-scrapping and nonattacking behaviors—not higher levels of self-processing. Their results and interpretations are thus irrelevant to the proposed importance of inner speech in self-awareness.

In addition, the authors’ view implies that since there seems to be self-recognition in fish, then fish are self-aware; fish do not engage in inner speech, thus inner speech is not required for self-awareness. Variations of this rationale have been shown to be flawed (2, 3): a) there is strong evidence that in humans, inner speech is indeed associated with processing of self-information (4, Table 1), as notably suggested by the fact that inner speech loss leads to self-awareness deficits (5); b) inferring that human and fish cognition can directly be compared, as done in the target paper, is uncalled for.

One last obvious, yet important observation, is that whatever results the authors obtained testing a total of 26 fish, they cannot be generalized to wild fish. The experimental fish were extensively exposed to their reflection in a mirror, anesthetized, photographed, and then reexposed to pictures of their faces. Clearly, wild fish will never encounter such unusual events in their natural habitat.
